# Impacts of Urban Sprawl on Soil Resources in the Changchun–Jilin Economic Zone, China, 2000–2015

**DOI:** 10.3390/ijerph15061186

**Published:** 2018-06-06

**Authors:** Xiaoyan Li, Limin Yang, Yongxing Ren, Huiying Li, Zongming Wang

**Affiliations:** 1 College of Earth Sciences, Jilin University, Changchun 130012, China; aurora_ylm@sina.com (L.Y.); 15271915264@163.com (Y.R.); lihuiyinghehe@163.com (H.L.); 2 Key Laboratory of Wetland Ecology and Environment, Northeast Institute of Geography and Agroecology, Chinese Academy of Sciences, Changchun 130102, China; zongmingwang@neigae.ac.cn

**Keywords:** urban sprawl, soil resources, soil quality, structure of the soil landscape, the Changchun–Jilin Economic Zone, China

## Abstract

The Changchun–Jilin Economic Zone (CJEZ) is one of the most rapidly developing areas in Northeast China, as well as one of the famous golden maize belts in the world. This is a case study to assess the impacts of urban sprawl on soil resources using remote sensing imagery and geographic spatial analysis methods. The common urbanization intensity index (CUII), soil quality index, and soil landscape metrics were calculated to reflect urbanization and the response of soil resource. Results showed that the area of soil sealing changed from 112,460 ha in 2000 to 139,233 ha in 2015, and in the rural region, the area occupied by urbanization nearly kept balance with the area of rural residential expansion. Urban land increased by 26,767 ha at an annual rate of 3.23% from 2000 to 2015. All seven soil types were occupied during the urbanization process, among which black soil ranked the highest (18,560 ha) and accounted for 69.34% of the total occupied area. Soils of Grades I (3927 ha) and II (15,016 ha) were 64.75% of the total occupied soil areas. Urban land expanded in an irregular shape and a disordered way, which led to an increasing large patch index (LPI) and aggregation index (AI), and a decreasing edge density (ED) and Shannon’s diversity index (SHDI) of the soil landscape in the study area during 2000–2015. According to the geographically weighted regression (GWR) model analysis, the *R*^2^ between the CUII and soil landscape metrics decreased from the LPI and ED to SHDI and in turn to AI. The local *R*^2^ between SHDI, ED, and CUII showed a gradient structure from the inner city to peri-urban areas, in which larger values appeared with strongly intensive urbanization in urban fringes. Soil sealing induced by urbanization has become a significant factor threatening soil, the environment, and food security. How to coordinate regional development and ensure the sustainability of the multiple functions of soil is a problem that needs to be taken into account in the future development of the region.

## 1. Introduction

Urbanization is a global process and is regarded as a necessary phase for most of the country’s development towards modernization [1]. The latest report from the United Nations showed that the global urbanization process of the rural population is still accelerating and the global urban population is expected to grow to 6.4 billion in 2050 from 3.9 billion in 2014 [2]. Urbanization inevitably leads to the expansion of urban land area. The area of global urban land quadrupled from 1970 to 2000 and the rate of expansion in developing countries even increased after 2000 [3,4]. According to Seto’s study, urban land areas in developing countries are projected to increase from 300,000 km^2^ to 1,200,000 km^2^ from 2000 to 2050 [5]. The growth of population and unprecedented urbanization not only created positive externalities through outstanding economic growth, but also greatly changed natural landscapes and brought enormous environmental and social impacts [6,7]. Much research has been made on the impacts of urban sprawl on landscape, air, water, biodiversity, as well as human health, but not enough attention has been paid on impacts to the structure and functions of soil resources [1,4,7,8].

During the urban expansion process, soils are often polluted with heavy metals, desurfaced, mixed, and compacted [9]. Many studies have been done on soil pollution with heavy metals (HMs) [4,10]. Recently, soil sealing and impermeabilization of soils resulting from urbanization have been attracting an increasing amount of interest [7,11,12]. Soil sealing was defined by Duley as a thin layer that limits infiltration through the soil surfaces [9]. Usually, the formation of soil sealing has natural and human factors [10]. Human activity, such as agricultural mechanization, and soil consolidation caused by long-term fertilization, has accelerated the natural soil sealing processes. Further, the spread of urban land leads to a permanent covering of soil surfaces in rural and urban regions. Because of the impermeabilization of soils, soil properties are changed, and exchange of gases, water, and energy are restricted. As a result, the multiple functions of soil, including its production, carrier, resource, habitat, and cultural functions, are obstructed [11]. Moreover, provided soil sealing happens, there is almost no possibility of restoration without a high cost. Soil sealing has been highlighted as one of the principal reasons of soil degradation and is of broad concern [10,11].

Recently, some monitoring schemes have been carried out. The soil sealing in Europe, East China, and the United States with rapid urbanization were studied and some valuable understandings were obtained [11,12,13,14]. Research indicated that soil sealing is a typical characteristic of urbanization [11]. Only for China, it has been estimated that there was a total soil sealing area of 25,523 km^2^ since 1990 and the increasing rate quickened after 2000 [12]. The results also indicated that fertile soils distributed in peri-urban areas were seriously jeopardized [13]. During 1990–2006, the area of soil sealing covering on agricultural land was summed up to be 11,890.7 km^2^ in 27 European Union member states [11]. Human construction activities interrupted the connection between soil and other ecosystem compartments and therefore influenced the soil function. Though there have been considerable publications on threats to soils, seldom have studies qualitatively described the changes in soil resources caused by rapid urbanization [10]. Discord between urbanization and soil resources is a global issue, which is particularly prominent in developing countries.

China entered the stage of rapid urbanization with rapid economic development in the late 1980s. The proportion of the urban population in the whole country increased from 10.6% to 49.9% in about 60 years since 1949 [15]. Northeast China is an old industrial region with a high level of urbanization. According to the statistics, the proportion of the urban population in Northeast China was 56.24% in 2010, which was above the Chinese average of 49.9% and the world average of 51.8% [16,17]. Rapid urbanization has led to many social and environmental problems including a loss of soil resources. Measuring the geographic traits of soil sealing in response to rapid urbanization can not only offer the basis for studying soil heterogeneity and ecological functions but also explore the interaction between soil landscape and human activities [18].

The Changchun–Jilin Economic Zone (CJEZ) is one of the regions with the most rapid urbanization in Northeast China, which is also one part of the famous black soil region and an important commodity grain base in China [19]. The CJEZ has experienced unprecedented economic development in recent years, especially after the implementation of the policy of Revitalization in Northeast China [20]. Since 2000, the gross domestic product (GDP) of the study area increased by 5.6 × 10^3^ billion RMB, and the non-agricultural population was raised by 496.6 × 10^3^ persons. As a result, the expansion of urban land quickened and led to a substantial increase in the area of soil sealing. Soil sealing has become a serious threat to the environment, food supply, and security. Thus, monitoring the process of soil sealing caused by urbanization and the impact of urbanization on soil resources are especially needed. 

Comprehensively using remote sensing and geographic information system (GIS) techniques, the objectives of this paper are (1) to analyze the urban sprawl in the CJEZ over the past 15 years, (2) to analyze the characteristics of soil types and soil quality occupied by expended urban land, and (3) to explore the impact of urbanization on the soil landscape.

## 2. Materials and Methods

### 2.1. Study Area Descriptions

**Figure 1:**
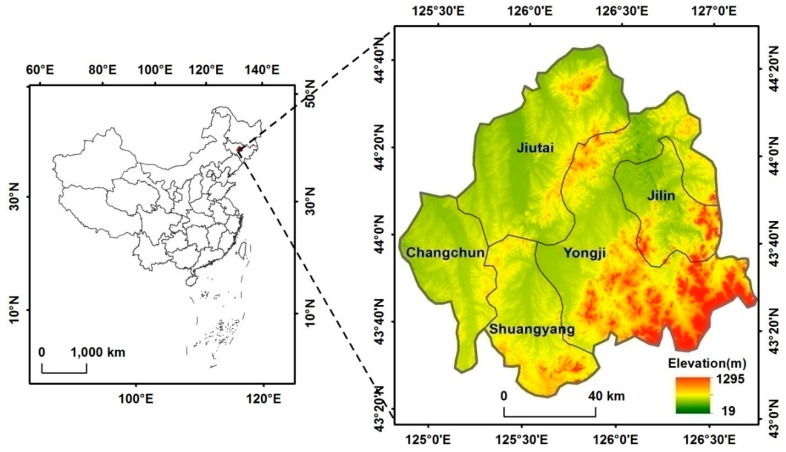
Location of the Changchun–Jilin Economic Zone (CJEZ).

The CJEZ is located in the middle part of the province of Jilin (124°50′16.8″ E–127°15′44.9″ E, 43°24′7.8″ N–44°22′55.2″ N) and includes Changchun, Jiutai, Jilin, Yongji, and Shuangyang (Figure 1), covering a total area of 12.9 × 10^3^ km^2^. The study area is characterized by the transition of a plain to a mountainous area, in which elevation changes from 19 to 1295 m from west to east. Accordingly, it has the continental and semi-humid climate of the cold temperate zone, which is characterized by cold winters and cool summers, with a mean annual precipitation and temperature of 570–700 mm and 4.5–4.8 °C, respectively, with rainfall concentrated mostly between May and September. The CJEZ is also in the famous black soil region.

### 2.2. Data Sources

#### 2.2.1. Land Cover Data

**Figure 2:**
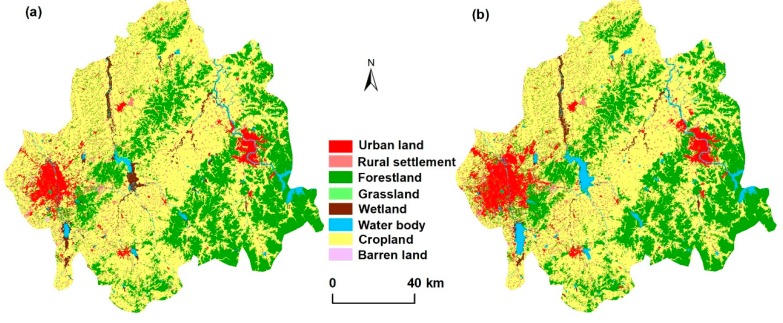
Land cover map of the CJEZ in 2000 (**a**) and 2015 (**b**).

Land cover maps of the study area were extracted from two sources: the Landsat Thematic Mapper (TM, six scenes) in 2000 and the Landsat Operational Land Imager (OLI, six scenes) in 2015 (Figure 2) with aquisition data between June and October. All images were preprocessed by band synthesis and geo-referenced to 1:100,000 topographic maps with an error within 1.5 pixel. For each image, the ground control points (GCPs) demanded more than 20 points. The land cover classification system includes eight classes (cropland, forestland, waterbody, grassland, urban land, barren land, wetland, and rural settlement). Cropland includes paddy fields and dry farming land. Forestland includes forests, shrubs, and orchards. Waterbody includes streams, rivers, lakes, reservoirs, ponds, and overflow land. Grassland is land covered by natural herbaceous plants with coverage greater than 5%. Urban land is the land used for residence, commerce, industry, recreation, and transportation in cities and towns. Barren land is the land with vegetation cover less than 5%. Wetland includes land with a permanent mixture of water and vegetation that covers extensive areas. Rural settlement consists of sparsely populated communities, away from densely populated urban centers. The images were classified into eight types of determined classes using a comprehensive method combined with the object-oriented classification method and the nearest neighbor classifiers [21] (using eCognition 8.64 software [18]) (Figure 2). First, the object-oriented method was used to segment the image into groups of homogeneous pixels so that the variability within the object was minimized [21]. Then, the compactness parameter was used to balance compactness and smoothness. After the objects in the images were segmented, image objects were classified into classes using nearest neighbor (NN) classifiers. To obtain the best interpretation, visual interpretation was carried out to confirm the extraction by inspecting high-resolution images available in Google Earth [21].

The accuracy of land cover has been assessed in previous studies [22,23]. In this study, additional validations including landscape records, GPS locations, and high-resolution Google Earth images of 280 ground truth points for each period were conducted to obtain specific classification accuracies of the land cover. The accuracies of land cover in 2000 and 2015 were 92.4% and 91.3%, respectively.

#### 2.2.2. Soil Data

**Figure 3:**
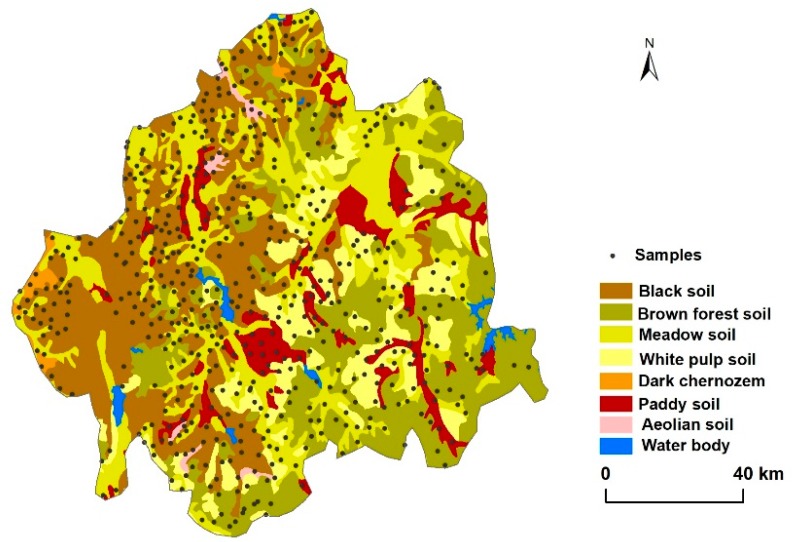
Distributions of soil types and sampling points.

Soil data included the soil type map and soil survey data. The digital soil type map with a scale of 1:500,000 was offered by the Soil and Fertilizer Station of Jilin province, China. The soil map was digitalized and updated in 2003. We merged the subcategories of soil classification into seven categories and analyzed the combined categories. In the study area, the main soil types are black soil (Luvic Phaeozem, Food and Agriculture Organization (FAO); 28.0%), meadow soil (EutricVertisol, FAO; 25.2%), brown forest soil (Haplic Luvisol, FAO; 24.2%), white pulp soil (MollicPlanosols, FAO; 13.6%), and paddy soil (HydragricAnthrosol, FAO; 8.2%) (Figure 3, Table 1). Approximate correspondence of soil type between Chinese soil classification and FAO reference soil taxonomy was conducted (Table 1).

The 619 sample data of the soil survey were obtained from the regional soil fertility investigation from 2006 to 2009 (Figure 3). Each sampling data was a mixture of five subsamples within 200 m^2^ from the soil surface (the depth was 0–20 cm). The data included the soil’s physical and chemical properties, as well as agricultural managements used. Samples were air-dried, crumbled, sieved through a 2 mm sieve and analyzed in the laboratory. The analytical methods for selected indicators are shown in Table 2.

### 2.3. Methodology

#### 2.3.1. Spatial Analysis of Urbanization

The common urbanization intensity index (CUII) is an effective method to reflect the dynamics of urban sprawl [25] and has a significant correlation with other widely used indices reflecting the intensity of urbanization, such as population density and the percentage of imperviousness [26]. We used 2 × 2 km of blocks as the basic unit to calculate the dynamic of urban sprawl via the following equation:

(1) $$CUII_{i}=\frac{A_{i,t+n}-A_{i,t}}{n\times WA_{i}}\times 100$$

where *CUII_i_* represents the urbanization intensity for spatial unit *i* during the time span *t* and *t* + *n*; *A_i,t+n_* and *A_i,t_* stands for the urban land area in the year *t* + *n* and year *t*, respectively, and $WA_{i}$ is the total area of the spatial unit *i* [26].

#### 2.3.2. Soil Quality Assessment

Soil quality reflects the soil’s ability to perform specific functions [27]. In order to estimate soil quality, seven indicators, i.e., soil organic matter (SOM), total nitrogen (TN), extractable phosphorus (AP), extractable potassium (AK), extractable Fe (AFe), cation exchange capacity (CEC), and pH, were collected to calculate the soil quality index [28,29]. All indicators were standardized by the standard scoring function (SSF)method [30], in which data were divided into two types according to their function on soil quality-upper limit type and peak limit type. Detailed SSF equations for the indicators are listed in Table 3. A principal component analysis (PCA) method was used to calculate the weights of each indicator. Soil quality index was then calculated via the integrated quality index (IQI). The IQI can be described with the following equation:

(2) $$\mathrm{IQI}=\sum_{i=1}^{n}w_{i}\times s_{i}$$

where IQI represent the integrated quality index, *w_i_* is the weight of the *i* factor for the soil property derived from the PCA method, and $s_{i}$ is the SSF score of *I* factor.

#### 2.3.3. Metric Analysis of the Soil Landscape

Metric analysis has been widely used to quantify the spatial structure and pattern of the landscape [31,32]. Guided by previous studies, four landscape metrics with low correlation at the landscape level were selected: the large patch index (LPI), the edge density (ED), Shannon’s diversity index (SHDI), and the aggregation index (AI) [10,32]. LPI is the area ratio of the largest patch and statistical unit, which reflects the degree of dominance. ED is calculated via dividing the total length of the patch boundaries by the total area, and increases when the patch shapes become irregular in the statistical unit. SHDI reflects the landscape heterogeneity and is especially sensitive to the non-equilibrium distribution of patch types in the statistical unit. When there is only one patch type in the statistical unit, SHDI is equal to 0; when the number of patch types increases or the area ratio of various patches is similar, SHDI increases accordingly. AI refers to the degree of non-randomness or aggregation of different patch types and the spatial configuration characteristics of landscape components [32]. These metrics characterize the size, composition, shape, and aggregation of the soil landscape.

In our study, the soil map was first masked by the urban land in 2000 and 2015, respectively. The soil map was then intersected with 2 × 2 km blocks to add the soil information to each block. Finally, each block was taken as a basically statistic unit to perform the metric analysis using ArcGIS 10.2 software (ESRI, Redlands, CA, USA). Expressions and ecological connotations of these metrics are shown in Table 4.

After the soil information in each block was acquired, change ratios of the selected metrics between 2000 and 2015 were calculated according to the following formula [10]:

(3) $$C_{i}=\frac{R_{2i}-R_{1i}}{R_{1i}}$$

where $C_{i}$ indicates the change ratio of landscape metrics in block *i*, *R*_1*i*_ is the metrics value of block *i* in 2000, and *R*_2*i*_ is the metrics value of block *i* in 2015.

#### 2.3.4. Geographically Weighted Regression Model

Soil types vary in different regions because of environmental heterogeneity. Traditional regression analysis has limitations in reflecting spatial constraints, whereas the geographically weighted regression (GWR) model can overcome such problems by considering locations [10,33,34]. GWR model is a local linear regression method and can generate local parameters to reflect spatial differences, including local *R*^2^, local model residual, and local coefficient. Therefore the complex spatial differences can be quantitatively simulated and mapped [10].

We selected the GWR model to explore the response of the soil landscape to urban expansion. The CUII of urban land was defined as dependent, and the change ratios of the soil metrics were defined as explanatory variables. The equation for the GWR model was as follows:

(4) $$y_{j}=\beta_{0}\left(u_{j},v_{j}\right)+\sum_{i=1}^{k}\beta_{i}\left(u_{j},v_{j}\right)x_{ij}+\varepsilon_{j}$$

where *u_j_* and *v_j_* are the spatial coordinates of the sample point *j*, $\beta_{0}\left(u_{j},v_{j}\right)$ is the intercept at the location *j*, $\beta_{i}\left(u_{j},v_{j}\right)$ is the estimated local coefficient of the independent variable *x_ij_*, and ε_*j*_ is the error term.

The model was performed in ArcGIS 10.2 software. Before the regressions, CUII and soil metrics data were normalized using the min–max standardized method.

## 3. Results

### 3.1. Urban Sprawl in the Past 15 Years

**Figure 4:**
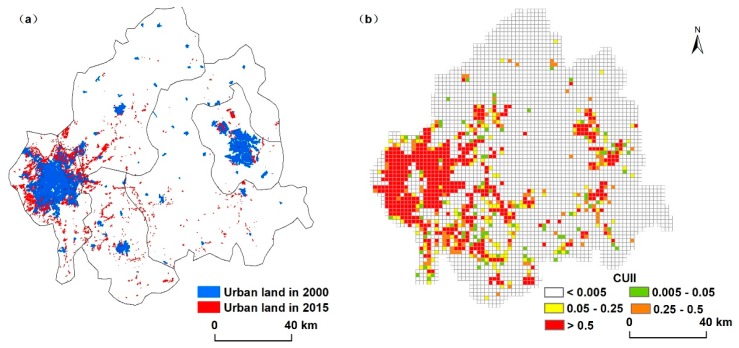
Distribution of urban land (**a**) and common urbanization intensity index (CUII) (**b**) in the CJEZ in 2000 and 2015.

The overall land use changes of the study area from 2000 to 2015 are listed in Table 5. Results indicated that in 2000, cropland was the dominant land use type, occupying more than 60% of the total area. In 2000–2015, the area of cropland and wetland decreased dramatically, while that of urban land and the water body increased significantly. The CJEZ had undergone considerable urban expansion since 2000 (Figure 4, Table 5). The area of urban land was 55,193 ha in 2000 and 81,960 ha in 2015, which increased by 26,767 ha in the past 15 years with a 1784 ha yearly increase (3.23%) (Table 5). Among the area of increased urban land, the proportion of cropland was as high as 92.87%.

The most significant expansion occurred in the peri-urban areas of Changchun and Jilin. In addition, the spatial patterns of urban expansion also reflected the heterogeneity in urbanization intensity within the CJEZ (Figure 4, Table 2). Changchun was the most urbanized area in this region and accounted for 62.58% of the total area with CUII greater than 0.5 (Table 6). Further, the urbanization intensity of Jilin and Shuangyang was higher than the surrounding areas.

### 3.2. Soil Types Occupied by Expanding Urban Land

Table 7 showed the soil area in 2000 and 2015. It can be seen from Table 7 that black soil, brown forest soil, and meadow soil were dominant soil types in the study area. Seven soil types were all involved in urban growth during the period of 2000–2015, but the pressure of urban sprawl was not equally distributed over all soil types. Black soil was the largest one occupying (18,560 ha) and accounting for 69.34% of the total occupied area. Meadow soil was another type of soil that was occupied more area and its contribution was 22.86%. Aeolian soil occupied less area among the seven soil types and its proportion was less than 1%. Though the brown forest soil distributed in large area, the occupied area was only 2.72% of the total occupied area and accounted for 0.24% in 2000. The loss of soil types may therefore represent loss of the whole biological communities unique to that soil type.

### 3.3. Quality Evaluation of the Sealed Soils

**Figure 5:**
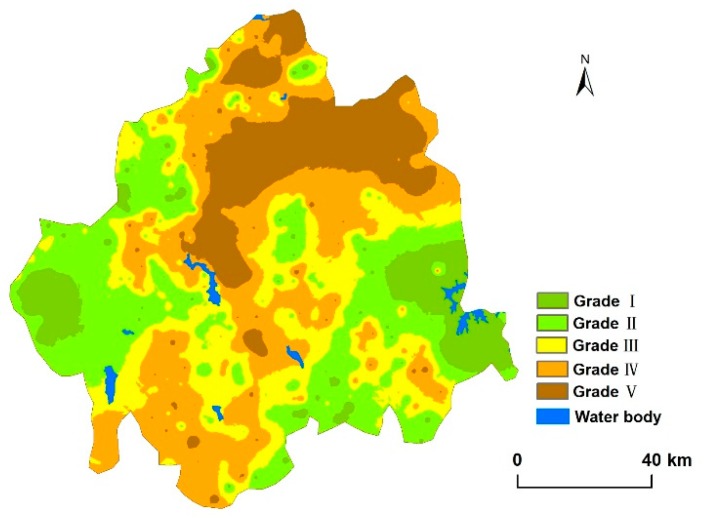
Distribution map of soil quality of the CJEZ.

According to the results of the IQI values for the soil quality assessment, the soil quality of the CJEZ was moderate, and Grade III was the dominant grade, covering an area of 1,020,913 ha (38.23% of the total area of soils) (Figure 5, Table 7). The soil area of Grades II and IV were 687,110 ha and 583,058 ha, accounting for 25.77% and 21.86% of the total area of soils, respectively. Only a small portion of soils were Grade I and Grade V, accounting for 9.15% (244,037 ha) and 4.94% (131,680 ha), respectively. Spatially, the highest values of IQI were distributed in the western and eastern areas, and the lower values (including Grades IV and V mainly) appeared in the middle part of the region. A downward trend was manifested from the west and east to the middle part of the study region.

Based on our analyses (Table 8), the total area of soils occupied by urban expansion in the past 15 years were up to 29,253 ha. An amount of 64.75% of the soil area occupied by urbanization were Grades I (3927 ha) and II (15016 ha) soils, which accounted for 13.42% and 51.33%, respectively. Grades IV (465 ha) and V (5725 ha) soils were 21.16% of the expanding urban area. The soil quality analysis of sealed soils indicated that the soils with high quality have a higher risk of being sealed.

### 3.4. Relationships between Soil Landscape and Urban Expansion

**Figure 6:**
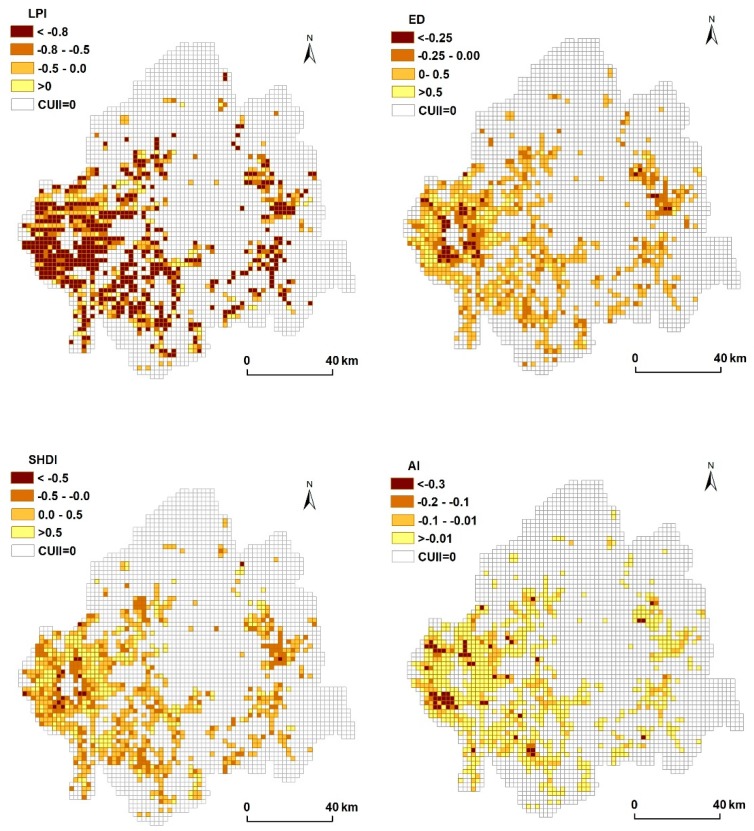
Change rates of soil landscape metrics during 2000–2015.

The change rates of soil landscape metrics were calculated on the condition of CUII > 0 using 2 km blocks, and the Jenks break point method was used to map the changes (Figure 6). 

It can be seen in Figure 6 and Table 9 that the mean change rate of the large patch index dramatically reduced from 2000 to 2015 when the soils become sealed and fragmented, resulting in more even patch areas in each block. The average change rate of SHDI and ED of the study area exhibited an increasing trend in 2000 and 2015, signifying increased instability, fragmentation, and irregularity of the soil landscapes. Moreover, the change rates of SHDI and ED represented a significant spatial structure. The inner city and suburban areas experienced an obvious circular structure with a decreased value in the inner city and increased value in the peri-urban area. AI was the only metric that decreased in the whole study area during the study span. Declines of AI denoted that soil landscapes became less dominant and aggregated. An obvious decrease in AI occurred in the west and north of Changchun, while the AI in the remaining areas did not obviously change. Among the four metrics, the change rate of SHDI was the largest and that of AI was the smallest. 

**Figure 7:**
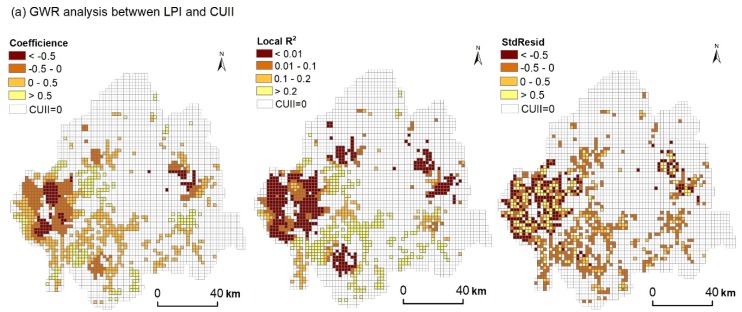

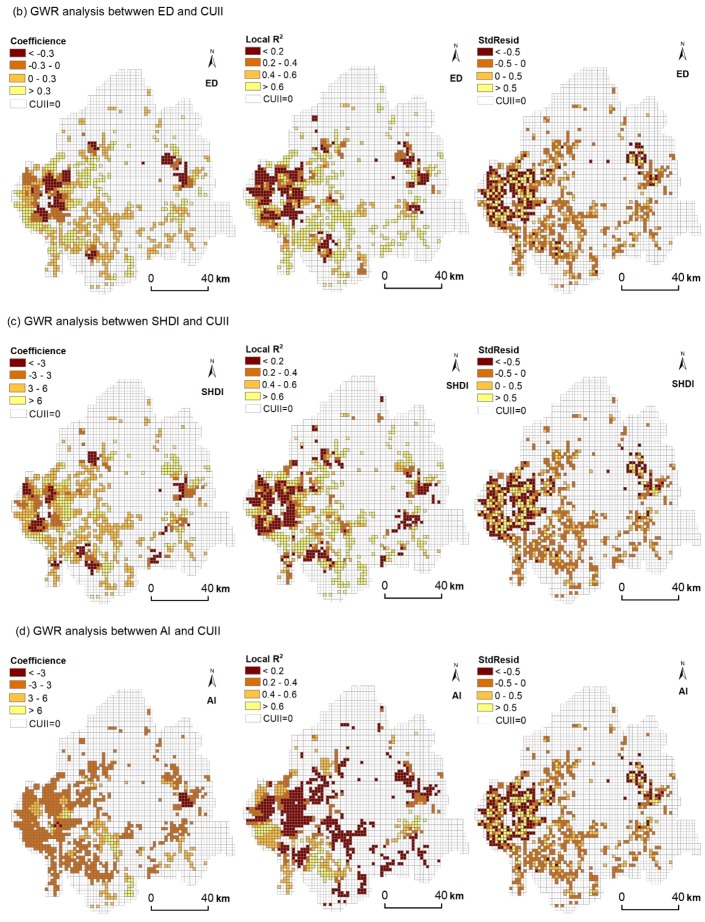
Local varying estimates of relationships between changes of (**a**) LPI and CUII, (**b**) ED and CUII, (**c**) SHDI and CUII, and (**d**) AI and CUII.

The GWR model was introduced to reflect the impact of intensity of urban sprawl on the soil landscape metrics (Figure 7). Some local varying parameters including the coefficient, adjusted *R*^2^, and standard residual (Stdresid) of each grid were produced by GWR, all exhibiting obvious spatial variability. A strong negative relationship between LPI and CUII (Figure 7a) and positive relationships between ED, SHDI, and CUII (Figure 7b,c) in blocks were reflected with intensive urbanization. For the LPI of soil, the obvious positive driving effect of CUII was mainly shown in the inner city of Changchun and Jilin. As for ED and SHDI, due to the inhomogeneity of urban sprawl in the direction, the CUII was anisotropic and thus led to the spatial anisotropic characteristics of ED and SHDI, especially for the local varying characteristic of Changchun. For AI, negative effects spread throughout the research area, and the correlation coefficient decreased from northeast to southwest (Figure 7d).

## 4. Discussion

### 4.1. Charicteristics of Soil Sealing in the Expanding Urban Land

Generally, urban sprawl appeared with the increasing population and intensification of economic activities. Urbanization means a conversion of natural or agricultural systems to impermeabilization, and this change was a leading cause of loss [34]. The conflicts between development and food security were always there. For urbanization in Northeast China, policy was one of the determinants in accelerating rapid urbanization [35]. The aim of establishing the CJEZ was, on the one hand, to make full use of the radiation driving effect of the central cities and to quicken regional development. On the other hand, the aim was to take the role of the satellite city around the large cities to alleviate the pressures on population and environment of big cities [31]. The study area witnessed unprecedented rapid urban land development since the implementation of the policy of revitalizing Northeast China. From2000 to 2015, the annual expanding rate of urban land in the CJEZ (3.23%) was higher than the mean value of the Northeast China (2.09%, during 2000 to 2010) and the whole country (2.75%, during 2000 to 2010; 2.15%, during 2006 to 2014), but lower than that of the middle region of China (5.44%, during 2000 to 2010; 4.68%, during 2006 to 2014) [16,36].

It can be seen from Figure 4 and Table 6 that, in the CJEZ, the areas with the largest expanding area of urban land (accounting for 35% of the total) and the highest expanding intensity of urban land (contributing 62.58% for the area of CUII > 0.5) were still found in Changchun (Figure 4). Changchun is located in the middle part of the Songneng Plain and the geomorphology consists of platforms (70%) and plains (30%). This means that human construction activities encountered fewer obstacles when the urban area was sprawling. At the same time, soils in Changchun consists mainly of black soil and meadow soil, which accounted for 49.78% and 29.06% of the farmland, respectively, and these two soil types were generally located in flat areas in close proximity to built-up areas in 2000. Therefore, these two soils lost a great amount of area due to urban sprawl. The results showed the largest area of occupied soils in this region were black soil (63.65% of the total occupied area) and meadow soil (22.86% of the total occupied area) (Figure 4). Once sealed, these areas would not likely be available for food production again. 

We also found that soils with the highest quality were distributed around Changchun (Grades I and II accounting for more than 55% of the total area) (Table 8). Despite the implementation of some farmland protection policies in this area, the rapid expansion of urban land is still consuming a large amount of high-quality cropland (Figure 5). A similar conclusion was drawn in the study conducted in the United States [37]. Consequently, the excessive loss of high quality soil continuously would definitely result in dramatically decreased food production and lead to a food security crisis. Coordinating the contradiction between rapid urbanization and cropland protection has become a problem that must be solved in regional sustainable development.

### 4.2. Impact of Urban Expansion on Soil Landscape

Landscape metrics have proven to be an effective way to investigate the evolution or dynamics of natural resources in response to urbanization [10,18,25]. Soils are dynamic components of terrestrial ecosystems, but the dynamics of the soil landscape were not given as the same concerns as the fate of others [10]. In our study, LPI, ED, SHDI, and AI were used to represent landscape characteristics of soil with urbanization. In sum, the dynamics of the soil landscape patterns were largely explained by the spatial characteristics of the spreading urban land. Urban land expanded in a disorderly way of strongly fragmented and irregularly shaped areas via interspersing among soil patches. As a result, strong relationships between the soil landscape pattern and the intensity indicator of urbanization were found in the rapid urbanization blocks, such as peri-urban areas (Figure 7). These indicated that the soil area decreased sharply, and even some soil types disappeared completely because of the expansion of urban land. All of this could lead to the abandonment of soils and result in a failure of their function in the ecological environment. 

Previous studies reflected the impact of urbanization on soil landscape via comparison or correlation analysis methods [10,38]. However, the kind of relationships in space between urbanization intensity and the soil landscape has not been clearly presented. We used GWR to describe the local varying parameter spatially and provide a quantitative contribution of urbanization to the change indifferent metrics of the soil landscape. Previous studies showed that the LPI and AI of the soil landscape will decline [10,39], while SHDI and ED will ascend with rapid urbanization [39], which is consistent with the statistic of the mean change ratio in our study. Nevertheless, in space, the SHDI and ED showed the gradient structure from the inner city to peri-urban areas, in which the increasing value appeared with strongly intensive urbanization (Figure 6 and Table 9).

According to Dietzel’s theory of urbanization, urbanization exhibits alternating cyclic patterns of diffusion and coalescence [40]. This theory implies that the threshold of the dynamics of landscape patterns exist as urbanization intensifies, which has been proven in previous studies of landscape fragmentation and landscape diversity [16,18]. Rui [10] compared 28 cities/counties with gradients in urbanization intensity to test whether or not the certain threshold also existed in soil landscape patterns, and the results showed that there was no threshold for soil landscape patterns. In our study, the expanding pattern of urban areas was a centralized model around the central city (Figure 4). Though all types of soil were occupied by urban sprawl, no soil type disappeared (Figure 4). Following the general model of urban development and Rui’s study result, some distinct soil types with unique physical structures and history of formation may be in danger of elimination, which would result in a substantial loss of below and above ground biodiversity.

### 4.3. Implications and Limitations

It is worth noting that various irreversible impacts on the structure and functions of soil resources brought by unprecedented rapid urbanization affected the soil’s capacity to deliver their diverse services and functions for human living and development. The dynamics trend reflected not only the tendencies of general urbanization in the CJEZ, but also mirrored the process of global urbanization [10]. In addition, the rapid urban sprawl in the CJEZ consumed a large number of good quality soils (Figure 5) that will never come back to agricultural use again. The researchers obtained essentially the same results in the United States, Europe, etc., in which high-quality soil resources were found to be in danger of great loss [11,41]. All of these highlighted the significance of soil sealing as a critical global issue. However, soil resources of developing countries experiencing fast urbanization have not received enough attention. Introducing the frameworks and quantitative analysis methods to conduct the changes of soil sealing under rapid urbanization should be pushed forward in the future.

There are still limitations associated with our study. We only chose 2 × 2 km as a basic unit to conduct the spatial analysis according to the comparative results of the existing research and did not quantitatively analyze the effects of different scales, though the scale effect was very prominent in the window analysis. Furthermore, limited by the sampling data, we simply analyzed the urbanization since 2000. Analysis of longer time sequences would give a better understanding of the dynamics of urbanization intensity and their impacts on the pattern, process, and functions of soil.

## 5. Conclusions

Soil sealing induced by urbanization has been considered as a serious threat to soil degradation, the environment, and food security. This study contributes to the quantitative analysis of urbanization and its influences on quantity, quality, and landscape of soil resources. The results are fundamental for governments for formulating policies against soil degradation within urban planning and regional development strategies.

Our study demonstrated the effectiveness of combining data of urban sprawl and change in soil resource in a quantitative way to describe the response of soil to rapid urban expansion. Results showed that the Changchun–Jilin Economic Zone witnessed a rapid urbanization process from 2000 to 2015. Sprawling urban land occupied a large number of high quality cropland in the suburbs, which was a significant factor for the change in soil landscape and jeopardized food production. How to coordinate regional development and ensure the sustainability of the multiple functions of soil, so as to guarantee agricultural production, needs to be determined for the sake of future development in the region.

## Figures and Tables

**Table 1:** Approximate correspondences of soil groups between Chinese soilclassification and Food and Agriculture Organization (FAO) reference soil taxonomy.

No.	Chinese Soil Classification System	FAO Soil Taxonomy	Area (ha)
1	Black soil	Luvic Phaeozem	371,645.18
2	Brown forest soil	HapicLuvisol	330,896.95
3	Meadow soil	EutricVertisol	345,896.88
4	White pulp soil	EutricPlanosols	185,757.96
5	Paddy soil	HydragricAnthrosol	111,647.78
6	Dark chernozem	Haplic Chernozems	11,265.07
7	Aeolian soil	Arenosol	10,280.84

**Table 2:** Methods used in laboratory analysis for selected indicators.

Indicator	Laboratory Analysis Method	Reference
Soil organic matter (SOM)	Potassium dichromate oxidation	[24]
Total nitrogen (TN)	Kjeldahl	[24]
Available phosphorus (AP)	Sodium bicarbonate extraction, colorimetric detection	[24]
Available potassium (AK)	Ammonium acetate extraction, flame photometer detection	[24]
Extractable Fe (Afe)	Flame photometer detection	[24]
Cation exchange capacity (CEC)	Ammonium acetate extraction	[24]
pH	Saturated soil paste	[24]

**Table 3:** The standard scoring function (SSF) method and the weights of indicators.

Indicator	FT	*x*_1_	*x*_2_	SSF	PCA	Weight
SOM (g/kg)	U(x)	13	50	$U\left(x\right)=\{\begin{array}{cc} 0.1 & x\leq x_{1}\\ 0.9\times \frac{x-x_{1}}{x_{2-x_{1}}}+0.1 & x_{1}<x<x_{2}\\ 1 & x\geq x_{2} \end{array}$	0.712	0.168
TN (g/kg)	U(x)	0.3	2.5		0.647	0.153
AP (mg/kg)	U(x)	1.5	4.5		0.612	0.144
AK (mg/kg)	U(x)	5	250		0.682	0.161
AFe (mg/kg)	U(x)	20	300		0.628	0.155
CEC (cmol/kg)	U(x)	10	35		0.656	0.148
pH	R(x)	5	7.05	$R\left(x\right)=\{\begin{array}{cc} 0.1 & x\leq x_{1}\\ 0.9\times \frac{x-x_{1}}{x_{2-x_{1}}}+0.1 & x_{1}<x<x_{2}\\ 1-0.9\times \frac{x-x_{1}}{x_{2-x_{1}}} & x\geq x_{2} \end{array}$	0.301	0.071

FT means function type; PCA means principal component analysis; *x*_1_ means lower limit; *x*_2_ means upper limit.

**Table 4:** Descriptions of the landscape metrics used in study.

Index	Equation	Ecological Connotation	Unit
Largest Patch Index (LPI)	$LPI=\frac{maxA_{ij}}{A}\times 100$	To indicate ratio of the largest patch area to total landscape area.	%
Edge Density (ED)	$\mathrm{ED}=\frac{E}{A}\times 10^{6}$	To denote the complexity of patch’s shape	m/ha
Shannon’s Diversity Index (SHDI)	$SHDI=-\sum_{i=1}^{r}p_{i}\ln p_{i}$	To indicate the landscape heterogeneity.	no
Aggregation Index (AI)	$AI=\frac{g_{ij}}{maxg_{ij}}\times 100$	To indicate the degree of aggregation among different landscape types.	%

**Table 5:** The conversion matrix of land use types from 2000 to 2015 (ha).

Land Use Type 2000	2015								
	Urban Land	Rural Settlement	Forestland	Grass-Land	Wet-Land	Water Body	Cropland	Barren Land	Total
Urban land	55,193								55,193
Rural settlement	598	54,564	45		10	50			55,267
Forestland	430	127	351,680		0		2920	9	355,166
Grassland	466		233	10		120	398		1226
Wetland	239	0	729		11,807	8030	2550	1	23,357
Waterbody	129		120		184	25,247	258		25,937
Cropland	24,858	540	13,644	7	2191	5073	729,900	6	776,219
Barren land	47		53	141	0	38	45	184	506
Total	81,960	55,258	366,504	157	14,192	38,558	736,069	201	1,292,900
Net change	26,767	−36	11,338	−912	−9165	12,622	−40,151	−306	
Change rate (%)	48.50	−0.07	3.19	−74.35	−39.24	48.66	−5.17	−60.39	
Urban land in 2015		598	430	466	239	129	24,858	47	
Change rate(%)		2.23	1.61	1.74	0.89	0.48	92.87	0.17	

**Table 6:** Average CUII in different regions.

CUII		<0.005	0.005–0.05	0.05–0.25	0.25–0.5	>0.5
Area		449,760,000	37,280,000	42,400,000	16,640,000	43,680,000
Proportion (%)		76.26	6.32	7.19	2.82	7.41
Changchun	Area (ha)	22,465,600	6,180,000	9,751,600	5,021,600	27,333,600
	Proportion (%)	5.00	16.58	23.00	30.18	62.58
Jilin	Area (ha)	45,554,000	4,583,600	3,842,400	1,766,800	3,756,000
	Proportion (%)	10.13	12.30	9.06	10.62	8.60
Jiutai	Area (ha)	115,872,000	7,164,400	7,394,800	3,790,000	6,704,400
	Proportion (%)	25.76	19.22	17.44	22.78	15.35
Shuangyang	Area (ha)	49,490,800	9,504,000	9,706,000	3,032,400	5,118,400
	Proportion (%)	11.00	25.49	22.89	18.22	11.72
Yongji	Area (ha)	186,924,800	8,736,800	11,117,600	2,982,400	523,200
	Proportion (%)	41.56	23.44	26.22	17.92	1.20

**Table 7:** Soil area in 2000 and 2015.

Soil Types	2000		2015		Occupied Soil		Occupied Soil/2000
	ha	%	ha	%	ha	%	%
Black soil	348,791.85	26.35	330,231.43	25.51	18,560.42	63.65	5.32
Brown forest soil	328,656.07	24.83	327,856.13	25.33	799.94	2.74	0.24
Meadow soil	333,590.70	25.20	326,923.64	25.26	6667.06	22.86	2.00
White pulp soil	181,886.81	13.74	180,676.78	13.96	1210.03	4.15	0.67
Paddy soil	109,682.42	8.29	108,411.35	8.37	1271.07	4.36	1.16
Dark chernozem	10,834.17	0.82	10,232.84	0.79	601.32	2.06	5.55
Aeolian soil	10,189.33	0.77	10,139.89	0.78	49.44	0.17	0.49

**Table 8:** Quality of soils urbanized in the CJEZ.

	Area of Soil		Occupied by Expanded Urban Land	
	Area (ha)	Proportion (%)	Area (ha)	Proportion (%)
Grade I	244,037	9.15	3,927.24	13.42
Grade II	687,110	25.77	15,016.32	51.33
Grade III	1,020,913	38.28	4,119.93	14.08
Grade IV	583,058	21.86	464.67	1.59
Grade V	131,680	4.94	5725.17	19.57

**Table 9:** Change ratio and the parameters of the soil landscape metrics in GWR.

	Ratio	AIC_c_	R^2^_CUII_	Adjusted R^2^_CUII_
LPI	−0.6386	−1346.71	0.6024	0.4925
ED	0.2170	−1429.56	0.5776	0.5049
SHDI	1.1619	−1326.56	0.5059	0.4390
AI	−0.0469	−1245.93	0.3500	0.3456
